# Initial Analysis on the Characteristics and Synthesis of Exopolysaccharides from *Sclerotium rolfsii* with Different Sugars as Carbon Sources

**DOI:** 10.3390/polym12020348

**Published:** 2020-02-05

**Authors:** Jia Song, Yu-Xiang Jia, Yan Su, Xiao-Yu Zhang, Lin-Na Tu, Zhi-Qiang Nie, Yu Zheng, Min Wang

**Affiliations:** 1 State Key Laboratory of Food Nutrition and Safety, Key Laboratory of Industrial Fermentation Microbiology, Tianjin Engineering Research Center of Microbial Metabolism and Fermentation Process Control, College of Biotechnology, Tianjin University of Science & Technology, Tianjin 300457, China; tjsongjia@tust.edu.cn (J.S.); 17829001@mail.tust.edu.cn (Y.-X.J.); susujiadeyanyan@163.com (Y.S.); 17320056773@163.com (X.-Y.Z.); tulinna@tust.edu.cn (L.-N.T.); 2 Key Laboratory of Chemical Biology and Molecular Engineering, Ministry of Education, Institute of Biotechnology, Shanxi University, Taiyuan 030006, China; nzqjason@126.com

**Keywords:** *Sclerotium rolfsii*, exopolysaccharides, carbon sources, characteristic, proteome

## Abstract

Scleroglucan is widely used in the food and chemical industries because of its good rheological property, stability, and emulsification activity. To investigate the influence of different carbon sources on the properties and synthesis of exopolysaccharides (EPS), the three EPSs (GEPS, glucose was used as the carbon source; LEPS, lactose was used as the carbon source; and SEPS, sucrose was used as the carbon source) were determined, respectively. It was found that the yield and viscosity of exopolysaccharides were different. When sucrose and glucose were used as the carbon sources, the viscosity and yield of EPS were both higher than lactose. The scanning electron microscopy (SEM) images showed that the three EPSs had different morphologies, but the monosaccharide analysis showed that they were all composed of glucose units. Fourier transform infrared spectroscopy (FT-IR) proved that there were no additional substituents for the three EPSs. Furthermore, the high performance liquid chromatography (HPLC) results showed that SEPS and LEPS had two fractions. Through the analysis of proteomics data, there were few differences in the metabolic pathways between GEPS and SEPS, but a significant difference between LEPS and SEPS. Our study provides a theoretical basis and reference for understanding the biosynthesis of exopolysaccharides and the development of different types of EPS products.

## 1. Introduction

*Sclerotium rolfsii* belongs to basidiomycetes, Umbelliferae, and Polyporus in fungi. The *S. rolfsii* exopolysaccharide, also known as scleroglucan, is produced by some species of *Sclerotium* [1,2,3]. It is considered to be a polymer that consists of β-(1→3)-linked glucose with a β-(1→6)-glycosyl branch on every third unit [4]. The exopolysaccharide is widely used in the food, chemical, and oil production industries because of its high water holding capacity [4], salt resistance [5], high temperature resistance [6], and shear resistance [7]. However, we found that by using different carbon sources, there were varied polysaccharide characteristics and yields, which aroused our interest in the study of the EPS structures and biosynthesis pathways.

Several studies have shown that the composition of the medium has a significant effect on the production of EPS. For example, Fariña et al. concluded that under the condition of 150 g/L sucrose, the yield of EPS was the highest [8,9]. Zhang et al. enhanced the EPS yield to 31.86 g/L and the fermentation broth’s viscosity reached 4500 mPa·S [10]. Schilling et al. described the effect of oxygen supply level on oxalic acid synthesis, and found that the higher the dissolved oxygen tension (DOT), the more oxalic acid was produced [11]. Gao et al. studied the effects of two feeding methods and confirmed that the feeding strategy with a constant flow rate could obtain higher biomass and EPS yield [12]. Schmid et al. studied scleroglucan and oxalate biosynthesis in *S. rolfsii* by using a transcriptomics approaches under scleroglucan-producing and non-producing conditions and identified the candidate genes [13]. These articles revealed the effects of different conditions on cell growth and the EPS production of *S. rolfsii*, but the characteristics of EPSs and how they are synthesized under different culture conditions are not clear. 

Given that the carbon source is the main factor that affects the characteristics of scleroglucan, this study explored the effects of different carbon sources on the characteristics of EPSs from *S. rolfsii* and a comparative proteomic analysis of *S. rolfsii* was performed to provide information that related to the pathway of EPS synthesis. This work provides a theoretical basis for further expanding the application of EPSs from *S. rolfsii* and for developing various EPS products in the food and biochemical industries. 

## 2. Materials and Methods

### 2.1. Materials and Reagents

The following materials and reagents were used: yeast extract, tryptone (OXOID Ltd, Basingstoke, Hampshire, England); anhydrous ethanol, phenol, trifluoroacetic acid and concentrated H_2_SO_4_ (Tianjin Chemical Reagent Plant II, Tianjin, China); glucose, lactose, mannose, maltose, fructose, sucrose, arabinose, and galactose (Tianjin Yaohua Chemical Plant, Tianjin, China); MgSO_4_·7H_2_O, NaNO_3_, K_2_HPO_4_, KCl, FeSO_4_, FeCl_3_ and citric acid (Tianjin Yaohua Chemical Plant, Tianjin, China); trichloromethane, acetonitrile-for HPLC (Tianjin Damao Chemical Reagent Factory, Tianjin, China); and n-butanol (Tianjin Jindong Tianzheng Fine Chemical Reagent Factory, Tianjin, China).

Except as specified, the reagents used were all pure analytical grade.

### 2.2. Fungal Strains and Growth Media

The fungal strain *S. rolfsii* ATCC 15205 was stored at the China Microbial Species Preservation Center. The strain was pre-cultured in potato dextrose liquid medium at 28 °C for 5–7 days under an agitated condition (220 rpm) to obtain seed liquid containing a large amount of mycelia. The seed liquid was inoculated into the fermentation medium with a 5% inoculation amount. The initial pH was 4.5–5.0, and the strain was cultured for 72 h to obtain EPS.

The fermentation medium was prepared in 250 mL conical flasks by dissolving carbon source (50 g/L), yeast extract (1 g/L), NaNO_3_ (2.25 g/L), K_2_HPO_4_ (2 g/L), MgSO_4_·7H_2_O (0.5 g/L), KCl (0.5 g/L), FeSO_4_ (0.05 g/L), and citric acid (0.7 g/L) in 100 mL of distilled water and sterilized at 115 °C for 30 min. The corresponding eight carbon sources were glucose, lactose, mannose, maltose, fructose, sucrose, arabinose, and galactose.

### 2.3. Rheological Property Measurement

The crude EPSs were extracted by the method of alcohol precipitation. First, 200 mesh filtration fabric was used for removing the mycelia. Then, the supernatant was precipitated (4 °C, overnight) with absolute ethanol (1:4) and the precipitate was collected by centrifugation (8000× *g*, 10 min). Finally, the crude EPSs were resuspended in deionized water and concentrated to 10 g/L by the way of evaporation concentration. The total sugar content of EPS was estimated by the phenol–sulfuric acid method with glucose as the standard. A BROOKFIELD viscometer (DV-II+) was used to measure the apparent viscosity at 12/s, 30/s, 60/s, and 100/s (25 °C).

### 2.4. Extraction and Purification of Exopolysaccharides

The crude EPSs were deproteinized by Sevage reagent (1-butanol/chloroform, *v*/*v* = 1:4) until a low amount of protein was left [14]. A Bicinchoninic Acid(BCA) protein assay kit was used to detect the protein content. Organic solvents were removed by the method of vacuum concentration. The small molecules were removed under flow water and deionized water for 48 h using dialysis bag (molecular weight (M_w_) cut-off: 3500 Da). The liquid in the dialysis bag was collected, and the EPS content was determined by the phenol sulfuric acid method.

### 2.5. Physicochemical Characterization of EPS

#### 2.5.1. Morphology Observation

The micro-structures of the three EPSs were observed by SEM (SU1510, Hitachi High Technologies, Tokyo, Japan) [15]. After freeze-drying, the EPSs were ground in a mortar. Then, the EPSs were coated with a layer of gold powder in a carbon coater at 10 Pa vacuum. Each sample was observed with 3.5, 7.0, and 10.0 K magnification.

#### 2.5.2. Chemical Composition Analysis

Several general physicochemical properties were tested: iodization reaction, FeCl_3_ reaction, Fehling’s test, phenol-sulfuric acid test, Coomassie brilliant blue reaction, Congo red reaction, and metal element analysis, according to the literature [16].

The composition and the content of metal elements in the EPSs were determined by inductively coupled plasma mass spectrometry (iCAP RQ ICP-MS) (Thermo Fisher, Shanghai, China). In brief, 0.2 g (accurate to 0.0001 g) of EPS was prepared in a 50 mL colorimetric tube, where 6 mL of nitric acid was added, and then hydrolyzed at 200 °C for 55 min. The solution was supplemented with ultra-pure water to 50 mL. The supernatant was used for mineral analysis. The content of mineral in the EPSs was calculated as follows:

$$\mathrm{Mineral}\;(\mathrm{mg}/g)=\frac{C*50}{m*1000}$$

where *C* is defined as the initial concentration of the samples (ppm), 50 is the volume of water (mL), and *m* is the weight of dried EPSs (g).

#### 2.5.3. Fourier Transform Infrared Spectra Analysis

The structural characteristics of the EPSs were evaluated by a Fourier transform infrared spectrometer (TENSOR 27, Bruker, Karlsruhe, Germany). Samples of EPS were freeze dried and ground evenly with dried KBr powder in a mortar. A 1 mg sample was mixed with 150 mg of dry KBr, and was pressed into a 1 mm pellet for the analysis. The resolution was 4 cm^−1^, and the scanning range was 4000–400 cm^−1^.

### 2.6. Molecular Weight Distribution and Monosaccharide Composition

#### 2.6.1. Molecular Weight Distribution

The purity of the EPSs was determined by high performance liquid chromatography (HPLC) (Agilent Technologies, Palo Alto, CA, USA) equipped with a TSK GEL G 5000 PWXL column (300 × 7.8 mm, Tosoh Corp., Tokyo, Japan) on an Agilent 1200 system and an refractive index detector (RID). The sample solution (1.0 mg/mL, 20 μL) was injected and eluted with distilled water at a flow rate of 0.6 mL/min with the temperature of 25 °C.

#### 2.6.2. Monosaccharide Composition

Monosaccharide composition was measured using the method described by Nie and Ma et al. [17,18] with slight modification. In brief, 2.0 mg of the dried EPS fractions were hydrolyzed with 2 mL of 2 M trifluoroacetic acid (TFA) at 120 °C for 3 h and then evaporated to dryness. Residual trifluoroacetic acid was removed with a small amount of methanol. Each sample was dissolved in deionized water and was filtered through a 0.22 μm filter membrane before analysis. Five standard sugars (D-Fucose, D-xylose, D-fructose, D-mannose, and D-glucose) were handled in the same way. Samples were analyzed by HPLC (Agilent Technologies, Palo Alto, CA, USA) equipped with an evaporative light scattering detector (ELSD) and a Prevail Carbohydrate ES column-250 mm × 4.6 mm 5 μm (Grace Davison Discovery Sciences, Baltimore, Maryland, USA). The gradient elution mobile phase was acetonitrile:water (75:25) at a flow rate of 1.0 mL/min. The column temperature was 35 °C, and the drift tube temperature was 90 °C. A 2.2 L/min carrier gas velocity was used.

### 2.7. Comparative Proteome Analysis

The mycelia of *S. rolfsii* cultured for 24 h with glucose, sucrose, and lactose, respectively, as carbon sources were collected in 1.5 mL centrifuge tubes and washed with sterile water three to five times. Then, the mycelia were frozen in liquid nitrogen for 10 min and stored in a refrigerator at −80 °C for proteome analysis. The Kyoto Encyclopedia of Genes and Genomes (KEGG) database was used to annotate the protein pathway. First, the KEGG online service tools was used to annotate the protein’s KEGG database description. Then, map the annotation result on the KEGG pathway database using the KEGG online service tools’ KEGG mapper. A two-tailed Fisher’s exact test was used to test the enrichment of the differentially expressed protein against all identified proteins. The pathway with a corrected *p*-value < 0.05 was considered significant. These pathways were classified into hierarchical categories according to the KEGG website. 

Tandem Mass Tags (TMT) kit was used to quantify the proteome. Proteins were filtered for statistical analysis (Student’s *t*-test) with a significance level of 0.05. A fold change (FC) >1.30 or <0.77 in protein abundance was further applied to define significantly changed proteins between the different carbon source samples statistically. Gene Ontology (GO) database and KEGG mappings were used to calculate the number of proteins in each term. The hypergeometric test was used to determine significantly enriched pathways.

### 2.8. Statistical Analysis

All experiments were conducted in triplicate. SPSS software (version 16.0, Chicago, IL, USA) was used for statistical analysis. The results were expressed as means ± standard deviations, and the *t*-test was performed at 5% confidence level. The statistical histograms and line charts were plotted by using OriginPro 9.0 (OriginLab Corporation, Northampton, MA, USA).

## 3. Results and Discussion

### 3.1. Rheological Comparison

**Figure 1:**
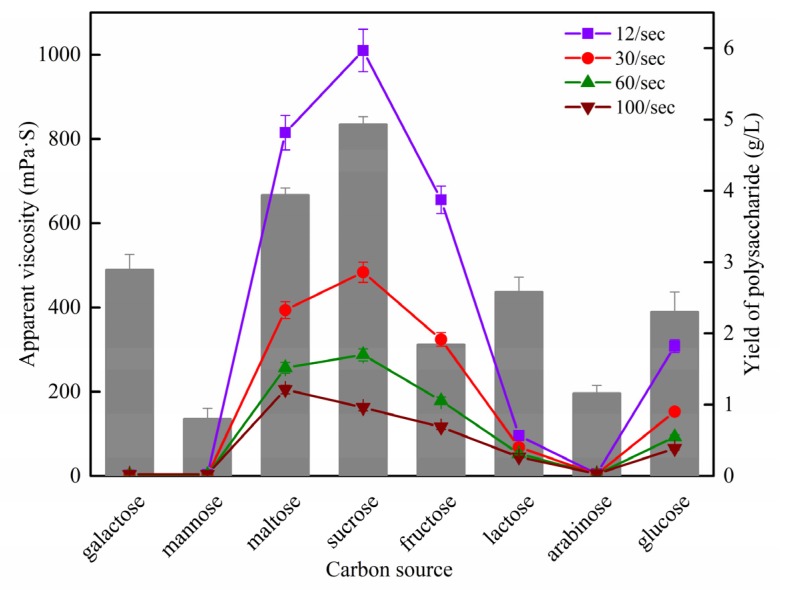
Comparison of the yield and rheological properties of exopolysaccharides (EPSs) from *S. rolfsii* ATCC 15205 with different carbon sources.

Different carbon sources can produce different results of EPSs, whether it is yield or viscosity. The yield and rheological properties of eight EPSs from different carbon sources are shown in Figure 1, where different carbon sources affected the apparent viscosity and yield of EPSs.

The yield of each EPS is shown as the histogram. When sucrose was used as the carbon source, the viscosity and yield of EPS was the highest, followed by maltose. When lactose and glucose were used as the carbon source, the EPS yield was moderate, with 2.58 and 2.30 g/L, respectively. Compared to each other, the yield of EPS from the highest to the lowest was sucrose, maltose, galactose, lactose, glucose, fructose, arabinose, and mannose.

However, at the concentration of 10 g/L, aqueous solutions of galactose-EPS, mannose-EPS, maltose-EPS, SEPS, fructose-EPS, LEPS, arabinose-EPS, and GEPS showed different viscosity changes, respectively. The maltose-EPS, SEPS, fructose-EPS, LEPS, and GEPS showed non-Newtonian pseudoplastic behavior (Figure 1), where the viscosity decreased with increasing shear rates. Pseudoplasticity might be related to the anisotropy of the rigid triple-helical structure frequently adopted in solution by this kind of polysaccharide [19]. In particular, the galactose-EPS, mannose-EPS, and arabinose-EPS showed little viscosity.

It is necessary for polysaccharides to show good viscosity and water holding capacity. From our perspective, different carbon sources might result in different molecular weights of EPSs or different grades of substitution with 1→6 linked side-chains and viscosity mainly dependent on the molecular weight of the EPS. As the basic chemical structure of scleroglucan should not be altered by the use of different carbon sources, the only effect might be in the different grade of substitution with 1→6 linked side-chains or the molecular weight. Therefore, in order to study the effect of different carbon sources on the characteristics of EPSs from *S. rolfsii* fermented, the representative carbon source needs to be selected for research. Taking the viscosity and the yield of EPS into account, we found that SEPS was the best choice. Though under the rotation rate of 100/s, the maltose-EPS had a better viscosity than SEPS. Additionally, although lactose is a disaccharide just like sucrose, the reason why there is such a big difference in viscosity and yield between them is worth exploring. Furthermore, GEPS, considered as scleroglucan, is widely used in factories, so we thought that it would be useful to compare it with SEPS or LEPS. Thus, SEPS, GEPS, and LEPS were selected for further study.

### 3.2. Scanning Electron Microscopy

**Figure 2:**
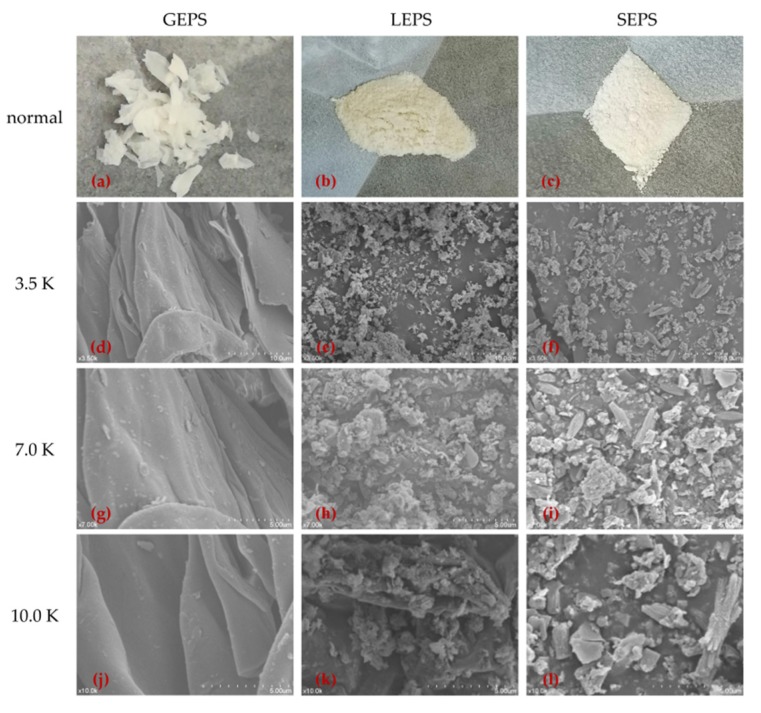
The appearance and scanning electron microscopy (SEM) images of EPSs at different magnifications (3.5, 7.0, and 10.0 K). (**a**) GEPS (normal); (**b**) LEPS (normal); (**c**) SEPS (normal); (**d**) GEPS (magnification, × 3500); (**e**) LEPS (magnification, × 3500); (**f**) SEPS (magnification, × 3500); (**g**) GEPS (magnification, × 7000); (**h**) LEPS (magnification, × 7000); (**i**) SEPS (magnification, × 7000); (**j**) GEPS (magnification, × 10,000); (**k**) LEPS (magnification, × 10,000); (**l**) SEPS (magnification, × 10,000).

The appearance and SEM results also showed the differences between GEPS, LEPS, and SEPS (Figure 2). After freeze-drying, the EPSs were ground in a mortar. The surfaces of the three kinds of EPS showed significant variations in size and shape when viewed with SEM. GEPS had a flat sheet structure with a small density and light weight. LEPS was slightly opalescent and granular, and SEPS was white granular. At magnifications of 3.5, 7.0, and 10.0 K, the SEM image showed that the inner part of GEPS was still a smooth layered structure. Meanwhile, under 10.0 K, the interior of LEPS (Figure 2k) and SEPS (Figure 2l) showed an irregular granular and rod-like structure, and the sample SEPS showed a clumpy surface with a small part of a chain-like structure.

### 3.3. Physicochemical Characterization of EPS

In order to compare the difference of the physical and chemical properties of these three EPSs, several tests were employed (Table 1). The iodization experiments showed no starch structure or reducing sugar in GEPS, LEPS, and SEPS. The Coomassie brilliant blue test showed that the three sugars did not contain any protein. The FeCl_3_ reaction test showed that the three sugars did not contain an enol structure. The phenol-sulfuric acid method was used to determine the EPS content.

**Figure 3:**
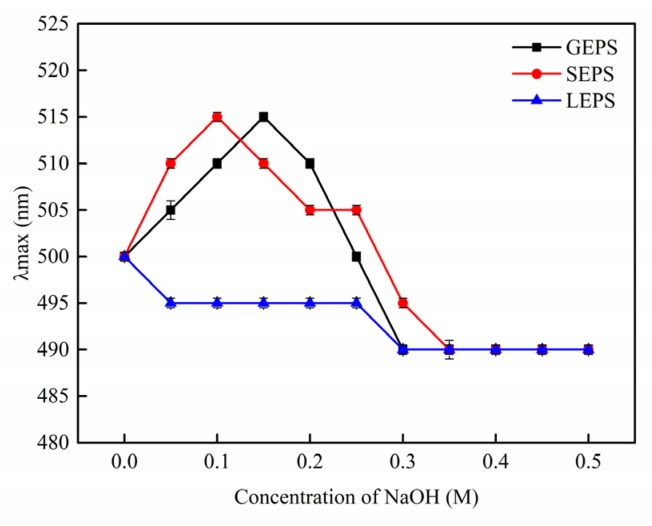
Maximum absorption (λmax) of Congo red + EPS at various NaOH concentrations.

The Congo red experiment results (Figure 3) indicate a triple helix structure in GEPS and SEPS, but not in LEPS.

Several common metal elements in EPS were analyzed by ICP-MS (Table 2). The contents of K+, Na^+^, Ca^2+^, Mg^2+^, and Fe^3+^ in GEPS were significantly different from those in LEPS and SEPS. The content of Ca^2+^ in GEPS was higher than that in LEPS and SEPS (P < 0.01), indicating that GEPS has a strong binding capacity to Ca^2+^. Conditions favoring scleroglucan production also increased the amount of oxalate secreted into the media [20], while when fructose was present in the medium, oxalic acid production decreased. Therefore, we deemed that oxalic acid coming from glucose binds to Ca^2+^, and precipitates with GEPS. LEPS and SEPS had higher binding capacities to K^+^, Na^+^, Mg^2+^, and Fe^3+^ than GEPS, therefore, we speculated that there may be more active groups in SEPS and LEPS, which gives them a good metal coordination ability.

### 3.4. Fourier Transform Infrared Spectra Analysis

**Figure 4:**
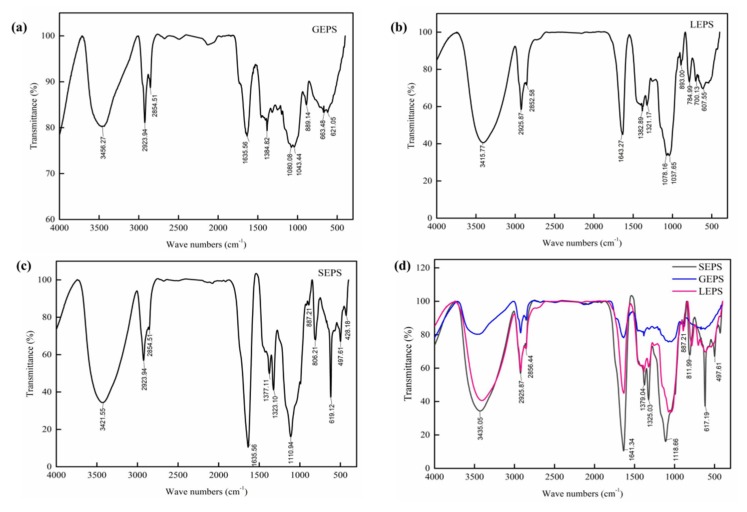
The FT-IR spectrum of (**a**) GEPS (glucose was used as the carbon source), (**b**) LEPS (lactose was used as the carbon source), (**c**) SEPS (sucrose was used as the carbon source) and (**d**) their comparison.

Results from the FT-IR spectra analysis revealed that the three EPSs had characteristic bands of polysaccharide (Figure 4). The O–H stretching vibration had a wide band at 3460–3415 cm^−1^ [21]. The weak bands at 2925 and 2852 cm^−1^ were the C–H stretching vibration absorption [22]. The absence of a band at 1728 cm^−1^ (C=O) indicates that there are no carboxylic sugars in this EPS [23]. The spectra of the three EPSs also showed two bands around 1540 and 1639 cm^−1^, corresponding to C–N and C–O stretching, that are related to the amide linkage of aminosugars in the polysaccharide structure. The bands around 1635–1643 cm^−1^ correspond to C=O, confirming that the sample contains aminosugars [23]. These observations are in agreement with the sugar compositional analysis [24]. The bands at 1500–1300 cm^−1^ are due to the angle-varying vibration of the CH_2_ bond [25]. The bands at 1080–1070 and 1043–1037 cm^−1^ can be attributed to C–O stretching vibration absorptions [26]. The band at 893–889 cm^−1^ is the characteristic absorption of β-glycosidic linkages [22]. The band at 806 cm^−1^ demonstrates the existence of an α-type glycoside bond [26]. Hence, we inferred that GEPS and LEPS are β-type polysaccharides, and there are both α-(806 cm^−1^) and β-(887 cm^−1^) glycosidic bond configurations in SEPS.

### 3.5. Molecular Weight Distribution

**Figure 5:**
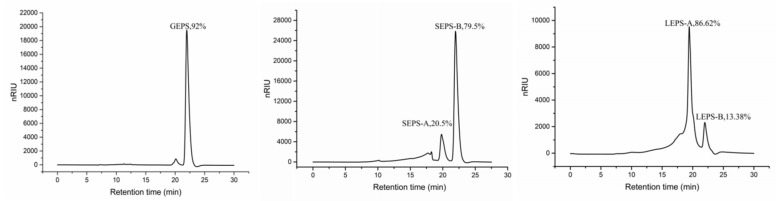
High performance liquid chromatography (HPLC) analysis of GEPS, SEPS, and LEPS.

The HPLC profiles of SEPS and LEPS presented two peaks, indicating that the EPS fractions of the two samples contained two major molecular weight distributions (Figure 5). Moreover, the retention time of the three EPSs was much the same. On the basis of these results, we did not think that the apparent viscosity differences were caused by the molecular weight, but that they might depend on the substitution with 1→6 linked side-chains. Additionally, the major component of GEPS, which had a higher percentage area (92%), was collected for monosaccharide analysis.

### 3.6. Monosaccharide Composition

It was shown that GEPS, LEPS, and SEPS are all glucans. A single peak was observed in the monosaccharide analysis, revealing that the EPSs contain a sole monosaccharide of glucose with no minor or trace component sugars (Table 3). Hence, the difference in viscosity was not caused by the monosaccharide composition. Furthermore, it is known that glucose is a monosaccharide, while sucrose is composed of glucose and fructose, and lactose is composed of galactose and glucose. Therefore, what caused such a huge difference is very interesting to explore further.

The biosynthetic route of scleroglucan in *S. rolfsii* is thought to follow the general anabolic pathway of glucose-based exopolysaccharides: glucose is taken up by glucose transporter(s) and phosphorylated to glucose-6-phosphate via a hexokinase reaction. After its isomerization to glucose-1-phosphate, UDP-glucose is formed via an UTP glucose-1-phosphate uridylyltransferase. A β-(1→3)-glucan synthase uses UDP-glucose as a substrate for polymer formation. Finally, a β-(1→3); (1→6)-glucosyltransferase is assumed to incorporate β-(1→6)-linked glucosyl side residues into the continuously elongating β-(1→3)-glucan backbone [20]. Thus, we speculate that glucose is involved in polysaccharide synthesis after entering the cells, while fructose and galactose are not directly involved in EPS synthesis. On one hand, the sucrose or lactose may be converted to glucose and participate in the glucose metabolism pathway as described above; on the other hand, they may be involved in the synthesis of the plasma membrane or cell wall.

Many EPSs synthesized by microorganisms have shown different structural and physicochemical properties under different culture media. For instance, EPSs synthesized by *Lactobacillus plantarum* CIDCA 8327 grown in skim milk and semi-defined medium (SDM) showed different structures. When grown in SDM, a heteropolysaccharide was produced that was composed mainly of glucose, glucosamine, and rhamnose, while the EPSs produced in milk were composed exclusively of glucose, indicating the influence of the sugar source [27]. The effects of culture conditions on the monosaccharide composition of *N. flagelliforme* EPS was significant. Changing the conditions of the carbon and nitrogen sources to increase the initial mole ratio of C/N obviously influenced the GDP–sugars synthetic pathway, especially mannose, by enhancing FBPase activity [28].

Based on the studies performed in bacterial, fungal, and microalgal species, EPS biosynthetic mechanisms are very complex, but the pathways and necessary enzymes (such as UDP-glucose pyrophosphorylase and UDP-glucose dehydrogenase) of typical EPS biosynthesis involved in the activation of the monosaccharides and conversion into sugar nucleotides are relatively conserved [28]. Therefore, we tried to find out the key enzymes or proteins that led to the difference in viscosity and yield between SEPS and LEPS by proteomics to provide a theoretical basis for the exploration of EPS synthesis.

### 3.7. Comparative Analysis of Metabolic Pathways

In organisms, different proteins coordinate with one another to perform their biological functions, which can be elucidated by pathway-based analysis [13,29,30]. Some proteins (and genes) that are involved in polysaccharide biosynthesis in bacteria have been identified, but the corresponding pathways in fungus are still unclear [31,32]. Different carbon sources lead to the expression of different proteins, which results in different EPS properties. To gain insight into how carbon sources flow in the EPS’s metabolism, we used mass spectrometry to quantify global changes in protein abundance and analyzed the pathway’s enrichment degree through the hierarchical clustering method based on Fisher exact test P value.

**Figure 6:**
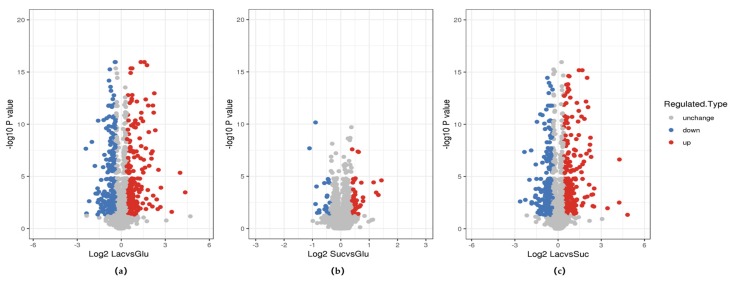
Quantitative volcanic map of differentially expressed proteins (**a**) group I, LEPS versus GEPS; (**b**) group II, SEPS versus GEPS; (**c**) group III, LEPS versus SEPS.

The threshold of differential expression was 1.3 times, the *t*-test p-value < 0.05 was used as the significant threshold, and the volcanic map of differentially expressed proteins are illustrated (Figure 6). About 260 proteins in the LEPS versus GEPS group (group I) were upregulated, and 273 proteins were downregulated (Figure 6a). About 268 proteins in the LEPS versus SEPS group (group II) were upregulated, and 265 proteins were downregulated (Figure 6c). Moreover, 46 proteins in the SEPS versus GEPS group (group III) were upregulated, and 29 proteins were downregulated (Figure 6b).

**Figure 7:**
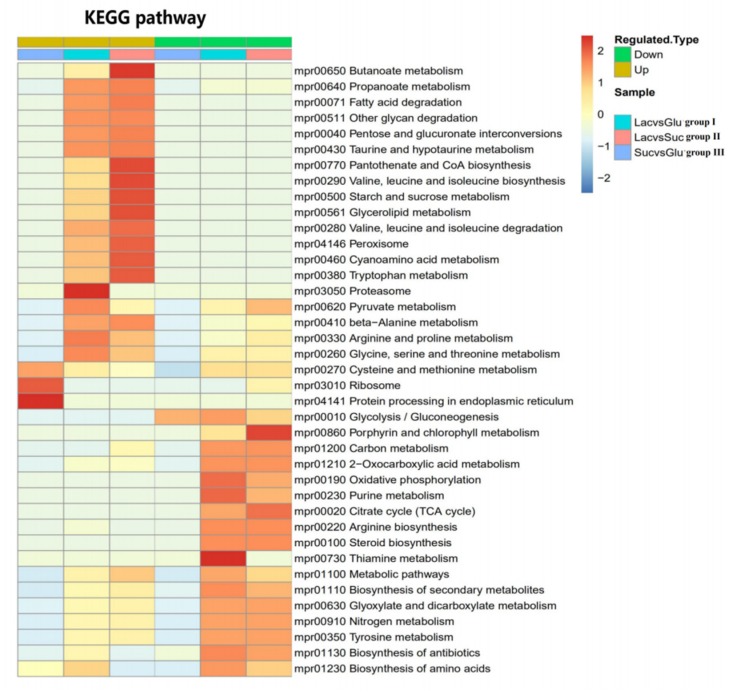
Cluster analysis heat map based on the enrichment of the Kyoto Encyclopedia of Genes and Genomes (KEGG) pathway.

Pathway enrichment was performed to analyze the potential mechanism of polysaccharide biosynthesis based on proteomic analysis in groups I/II/III (Figure 7).

In group I, significantly downregulated pathways in the LEPS-GEPS comparison group included glyoxylate and dicarboxylate metabolism (map 00630), tricarboxylic acid cycle (map 00020), glycolysis/gluconeogenesis (map 00010), and oxidative phosphorylation (map 00190), which are related to carbohydrate metabolism and energy metabolism. The downregulation of these pathways led to the inhibition of mycelia growth as well as the polymerization of EPSs [33]. The other pathways such as other glycan degradation (map 00511), starch and sucrose metabolism (map 00500) were upregulated in LEPS. The α-glucosidase, β-glucosidase, and glucoamylase in map 00500 were upregulated, which may result in the low molecular weight LEPS and reduce its rheological properties.

In group II, the results were basically similar to group I. The downregulated pathways such as tricarboxylic acid cycle (map 00020), glycolysis/gluconeogenesis (map 00010), and oxidative phosphorylation (map 00190) nearly matched the downregulated pathways in group I. The upregulated pathways were related to carbohydrate metabolism and the metabolism of cofactors and vitamins such as butyrate metabolism (map 00650), pantothenic acid and coenzyme A biosynthesis (map 00770), and starch and sucrose metabolism (map 00500) [34].

In group III, the upregulated pathways in SEPS were related to translation, folding, sorting, and degradation and amino acid metabolism: ribosome metabolism (map 03010), endoplasmic reticulum protein processing (map 04141), and cysteine and methionine metabolism (map 00270). These pathways were thought to be related to the growth of the mycelia. Ribosomal proteins (RPs) are components of ribosomes that are involved in translation and thus indispensable for growth, cell division, and metabolism [35]. Additionally, we found that the amount of mycelia of SEPS was more than GEPS to some extent, which positively correlated with the result of EPS yield. The downregulated pathway was the glycolysis/glycogenesis pathway (map 00010).

## 4. Conclusions

According to the difference in viscosity and yield, the EPSs of *S. rolfsii* fermented with glucose, sucrose, and lactose as carbon sources were screened and their structures were preliminarily characterized. What is interesting is that while the GEPS, LEPS, and SEPS were all composed of glucose, their rheological properties were quite different. Microscopic morphology and infrared spectroscopy analyses showed the differences between the three EPSs. Furthermore, the differences in the metabolic processes of *S. rolfsii* under the three carbon sources were explored. Although we explained the changes of some metabolic pathways in the growth process of *S. rolfsii* under different carbon source fermentation conditions, the specific metabolic process is still unclear, and more experiments are needed to verify the key steps of the EPS synthesis.

In summary, the initial structure analysis on EPSs combined with proteomic analysis provided important insights into the effects of carbon sources on microbial EPS fermentation. Manipulation of medium composition, especially carbon source, can not only increase the EPS yield of *S. rolfsii*, but also influence its structure and viscosity characteristics. This strategy can also be applied to develop new polysaccharide products after further exploration of its activity.

## Figures and Tables

**Table 1:** Comparison of physicochemical properties of EPSs.

Method	GEPS	LEPS	SEPS
Iodination test	−	−	−
Fehling’s test	−	−	−
Phenol-sulfuric acid test	+	+	+
Coomassie brilliant blue test	−	−	−
FeCl_3_ test	−	−	−

Note: The expression of “−” means the reaction was negative, the expression of “+” was positive.

**Table 2:** Composition and amounts of metals in EPSs.

Samples			Metals (mg/g)		
	K^+^	Na^+^	Ca^2+^	Mg^2+^	Fe^3+^
GEPS	0.66 ± 0.12 ^a,b^	14.68 ± 1.25 ^a,b^	23.06 ± 2.04 ^a,b^	1.95 ± 0.31 ^a,b^	0.89 ± 0.22 ^a,b^
LEPS	52.37 ± 3.65 ^c^	19.65 ± 2.32 ^c^	2.03 ± 0.43	16.87 ± 2.13 ^c^	12.74 ± 1.55 ^c^
SEPS	64.57 ± 3.07	27.54 ± 2.04	1.97 ± 0.42	20.48 ± 1.02	15.37 ± 1.24

Note: ^a^ indicates the mean values differ significantly in the group of GEPS and LEPS (P < 0.05), ^b^ indicates the mean values differ significantly in the group of SEPS and GEPS (P < 0.05), and ^c^ indicates that the mean values differ significantly in the group of LEPS and SEPS (P < 0.05).

**Table 3:** Monosaccharide composition of EPSs.

Standard Samples	D-Fucose	D-Xylose	Fructose	Mannose	D-Glucose
Retention time (min)	5.679	6.767	7.231	8.264	9.157
GEPS	-	-	-	-	+
SEPS-A	-	-	-	-	+
SEPS-B	-	-	-	-	+
LEPS-A	-	-	-	-	+
LEPS-B	-	-	-	-	+
